# Dietary fiber from burdock root ameliorates functional constipation in aging rats by regulating intestinal motility

**DOI:** 10.3389/fnut.2025.1550880

**Published:** 2025-03-24

**Authors:** Liya Mo, Kaiyang Ma, Ying Li, Jiangfeng Song, Qiqi Song, Ling Wang

**Affiliations:** ^1^Department of General Medicine, The First Affiliated Hospital of Soochow University, Suzhou, Jiangsu, China; ^2^Department of Chinese Medicine, Suzhou Vocational Health College, Suzhou, Jiangsu, China; ^3^Institute of Agro-Product Processing, Jiangsu Academy of Agricultural Sciences, Nanjing, China

**Keywords:** dietary fiber, burdock root, functional constipation, the aging, nutritional strategy

## Abstract

Functional constipation is a common gastrointestinal health issue among the aging population. Dietary fiber supplementation is widely recognized as a first-line strategy for constipation. However, the effectiveness of dietary fiber in practical applications remains unsatisfactory, and dietary fibers from different sources are believed to possess varying physiological activities in alleviating constipation. Burdock root is a vegetable rich in dietary fiber. In this study, loperamide was used to induce functional constipation in aged rats. Doses of 3 mg/kg·bw·day and 1.5 mg/kg·bw·day of dietary fiber from burdock root were used to intervene in functional constipation in aged rats. Research indicated that dietary fiber from burdock root enhanced intestinal motility to ameliorate functional constipation in aging rats. This effect may involve several mechanisms, including repairing the intestinal barrier, regulating intestinal hormones, and providing anti-inflammation and antioxidation. Our findings provide a theoretical basis for the potential mechanism by which burdock root dietary fiber can ameliorate functional constipation. It is expected to serve as a natural functional food to combat functional constipation in the aging population.

## 1 Introduction

Constipation is a gastrointestinal disorder characterized by infrequent bowel movements, difficulty in defecation, and dry stool (1). Functional constipation (FC) is a persistent condition that excludes organic diseases and drug factors. It is common among the aging population and significantly associated with colon cancer, hepatic encephalopathy, and stroke (2). The primary causes of FC in the aging population include a decelerated metabolism, gastrointestinal motility dysfunction, and pelvic floor dysfunction (3). Current treatments involve empirical approaches, such as dietary interventions, habit training, and pharmacotherapy (4). While drugs are typically effective, long-term use puts stress on the liver and kidneys in aging patients, and habit training shows limited effectiveness in severe cases. Therefore, identifying safe and effective interventions for aging patients is essential.

In clinical practice, increasing dietary fiber intake is considered a first-line treatment for constipation (5). Dietary fiber found in whole grains, fruits, and vegetables offers a safer alternative to drug therapy for aging patients (6). As an indigestible carbohydrate with beneficial physiological effects, insufficient dietary fiber is associated with a higher risk of gastrointestinal disorders such as FC (7). Dietary fiber is classified into soluble and insoluble types, each having distinct effects on constipation. Insoluble fiber increases intestinal volume and osmotic pressure, while soluble fiber stimulates intestinal motility through metabolites, such as short-chain fatty acids released by gut flora (8). However, in aging patients, excessive supplementation of either type of fiber can lead to complications such as irritable bowel syndrome or gastrointestinal distress (9). Thus, the balanced total dietary fiber intake is essential for managing FC in aging people.

The anticonstipation effects of dietary fiber involve complex mechanisms, including enhanced intestinal motility. Studies indicate that dietary fiber significantly increases intestinal propulsion in rats; however, the exact effects on intestinal motility remain underexplored (10, 11). Intestinal hormones, neurotransmitters, and interstitial cells of Cajal (ICCs) play essential roles in regulating intestinal movement, which may be influenced by dietary fiber (12). During this process, intestinal neurotransmitters and the number of interstitial cells of Cajal (ICCs) will also influence intestinal slow-wave motion (13). Additionally, oxidative stress and inflammation caused by constipation might disrupt intestinal homeostasis and potentially interact with the effects of dietary fiber (14).

Burdock root is a popular vegetable in many countries and regions. Interestingly, studies have shown that burdock root has the potential to promote intestinal transport and ameliorate gastrointestinal diseases (15, 16). However, burdock root is rich in dietary fiber, and the effect of dietary fiber from burdock root (BDF) on FC in the aging stage has rarely been studied. Given that dietary fiber’s structure varies by source and influences its function, and considering that fibers from different sources have different effects on constipation (17), the practical application of dietary fiber as a treatment for severe constipation is not satisfactory. Therefore, this study aims to explore the anticonstipation effects and potential mechanisms of dietary fiber from burdock root in aging rats with FC.

## 2 Materials and methods

### 2.1 Chemicals and reagents

Protease and phosphatase inhibitors and Bradford Protein Assay Kit were purchased from Beyotime (Haimen, China). The total protein assay kit using the standard BCA method, phenol red, and activated carbon was purchased from Nanjing Jiancheng Bioengineering Institute. High-purity total RNA extraction kit; ChamQ Universal SYBR quantitative polymerase chain reaction (qPCR) Master Mix and reverse transcription-polymerase chain reaction (RT-PCR); and HiScript III RT SuperMix for qPCR (+gDNA wiper) were purchased from Vazyme Biotech (Nanjing, China). Rat Reactive Oxygen Species (ROS) Enzyme-Linked Immunosorbent Assay (ELISA) Kit was purchased from King Biotech Life Sciences (Mukund Nagar, India). Rat Acetylcholine (ACh) ELISA Kit, Rat Vasoactive Intestinal Peptide (VIP) ELISA Kit, Rat Gastrin (Gas) ELISA Kit, Rat Motilin (MTL) ELISA Kit, Rat Catalase (CAT) ELISA Kit, Rat Superoxide Dismutase (SOD) ELISA Kit, Rat glutathione peroxidase (GSH-Px) ELISA Kit, Rat Choline Acetyltransferase (ChAT) ELISA Kit were purchased from Gelatins (Jiangxi, China).

### 2.2 Preparation of dietary fiber from burdock root

*Arctium lappa* L (burdock) was harvested in Feng County, Xuzhou City, China. Burdock root powder was obtained by the ultrafine grinding method. Burdock root powder and water were mixed in a ratio of 1:20. The pH was adjusted to 6.0 and 0.6% (w/w) of *α*-amylase was added at 60°C for 40 min. Then the pH was adjusted to 4.5 and 1% (w/w) of glucoamylase was added at 60°C for 40 min. Next, the pH was adjusted to 6.0 and 4.0% (w/w) of papain was added at 50°C for 60 min and stopping the enzyme at 100°C for 5 min. Then, four times the volume of anhydrous ethanol is added at 4°C overnight. The precipitation was collected by centrifuging at 5,000 *g* for 10 min, with repeated cleaning twice. Finally, the dietary fiber powder derived from burdock root (BDF) was obtained through freeze drying. The selection of the enzymatic hydrolysis conditions was based on preliminary experimental results (18). Under the specified temperature, pH, and time conditions, the substrate can undergo thorough enzymatic hydrolysis, facilitating the more efficient removal of starch and protein.

### 2.3 Animals and experimental design

All 12-month-old male Wistar rats were purchased from Beijing Vital River Laboratory Animal Technology Co., Ltd (Nanjing, China). The experimental animal quality certificate number is SCXK (Beijing) 2021-0006. The rats were kept in the Animal Resource Center for 6 months. The rats ate and drank freely in a room with an ambient temperature of 22 ± 2°C and a light–dark cycle time of 12:12 h.

A total of 49 Wistar rats were divided into a control group (7 rats) and a model group (42 rats). The model rats received loperamide (1.5 mg/kg·bw·day) for 21 days to induce FC. Then, the model rats were further divided into four groups: model (FC), low-dose BDF (BDF-L), high-dose BDF (BDF-H), and Maren pill (MP). The rats in the control group (CON) and the FC group were given continuous intragastric administration of normal saline, BDF-L, and BDF-H were given daily administration of BDF. Furthermore, 3 mg/kg·bw·day was used as the BDF-H group, 1.5 mg/kg·bw·day was used as the BDF-L group, and MP was selected as the positive drug with the dose of 3 g/kg·bw·day. The intervention lasted 21 days, during which body weight, food intake, fecal count, and fecal weight were monitored daily. After the experiment, the rats’ tissues were stored at −80°C.

Ethical approval statement: This study was conducted in strict accordance with the guidelines of the Institutional Animal Care and Utilization Committee of Nanjing Agricultural University. The ethical approval number for the use of animals is njau 20220830161.

### 2.4 Determination of fecal water content

Fresh feces of rats were weighed and dried in the oven to a constant weight with the temperature set at 105°C. The calculation formula is as follows: Fecal water content (%) = [(fresh fecal weight − dry fecal weight)/fresh fecal weight] × 100%.

### 2.5 First black stool excretion

Approximately 10% activated carbon was given to rats by gavage. The rats were kept alone in cages. The excretion time of the first black stool was recorded, and the feces were collected within 6 h.

### 2.6 Analysis of intestinal propulsion rate

Before the end of the experiment, each rat fasted for 24 h. Then the rats were administered with phenol red suspension. After waiting for 30 min, the rats were anesthetized, and the small intestine from the pylorus to the cecum was collected. The intestinal propulsion rate of rats was calculated, that is, the ratio of the length of phenol red advancing in the small intestine to the total length of the entire small intestine.

### 2.7 HE staining

The intestinal tissue of rats was fixed in 4% paraformaldehyde for 48 h and dehydrated with different concentrations of ethanol. After curing, the slices were cut into 5-μm slices with a microtome, which were pasted on a slip-proof slide to prepare specimens for baking at 65°C for 1 h. When the dyeing is over, the sections were dehydrated with an alcohol gradient and made transparent with xylene. Finally, it was sealed with neutral balsam. The image is examined under a light mirror, and the image is collected and analyzed. All photographs were taken with an optical microscope (ECLIPSE 80i; Niko, Tokyo, Japan) with 20× magnification.

### 2.8 Real-time quantitative PCR experiment

The total RNA of rat intestinal tissue was extracted with a high-purity total RNA extraction kit, and the following operations were carried out according to the instructions. In each sample, GAPDH was used as an internal reference, and differences in total RNA were normalized. PCR reaction primers were synthesized by GenScript USA, Inc., and the primer sequences are listed in Table 1.

**Table 1:** Target genes and primers used in RT-qPCR experiments.

Target gene	Primer	Sequence (5′-3′)
GAPDH	Forward Reverse	AGGTCGGTGTGAACGGATTTTG GGGGTCGTTGATGGCAACA
SOD	Forward Reverse	CATTCCATCATTGGCCGTACT CCACCTTTGCCCAAGTCATC
GSH-Px	Forward Reverse	GCGCTGGTCTCGTCCATT TGGTGAAACCGCCTTTCTTT
Zo-1	Forward Reverse	CGATTGCTGATGTTGCCAGA CGATCGACTGCATTTGGTGT
Occludin	Forward Reverse	TGACCAGTGACATCAGCCAT TGCATCTCTCCGCCATACAT
AQP1	Forward Reverse	CCTGCTGGCCATTGACTACA TGGTTTGAGAAGTTGCGGGT
AQP2	Forward Reverse	TTGCAGGAACCAGACACTTG GCGGAGACGAGCACTTTTAC
AQP3	Forward Reverse	ACTCCAGTGTGGAGGTGGAC GCCCCTAGTTGAGGATCACA
AQP4	Forward Reverse	TGGTCCTCATCTCCCTCTGCTT AACCGTGGTGACTCCCAATCCT

### 2.9 Data statistics

All data were expressed as a mean ± standard deviation (SD), and Student’s *t*-test and analysis of variance (ANOVA) were used. In all studies, *n* represents the sample size per group, and a *p*-value of <0.05 is considered statistically significant.

## 3 Results

**Figure 1:**
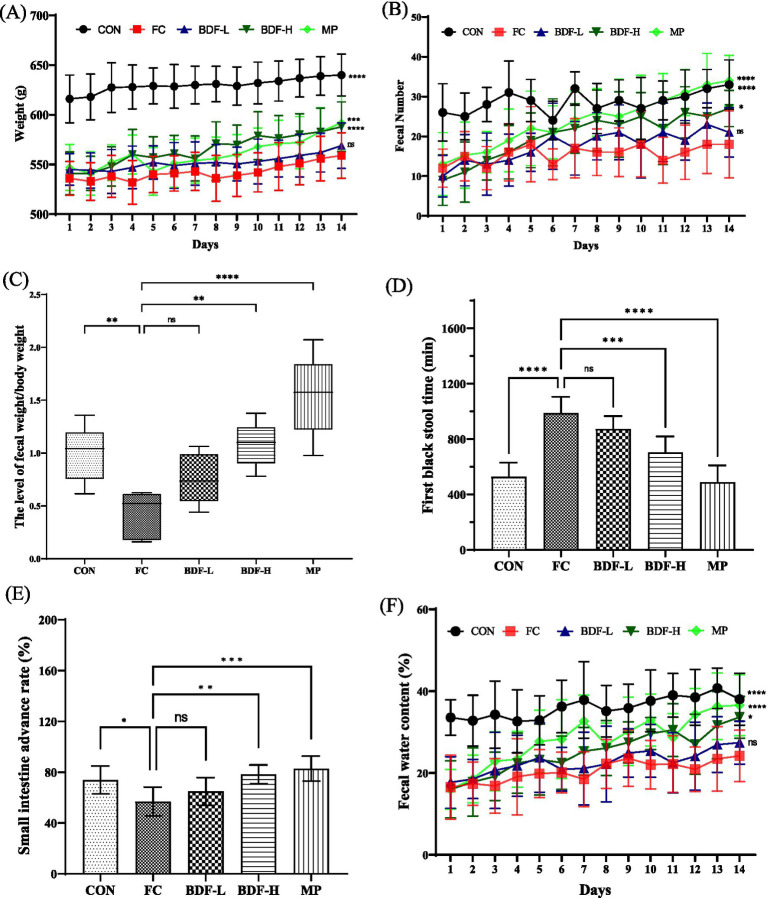
BDF moistened bowel laxation and enhanced intestinal motility. **(A)** Weight and **(B)** fecal number that were detected daily. **(C)** Fecal-to-body weight ratio. **(D)** First black stool time that was analyzed on day 14. **(E)** Small intestine propulsion rate was analyzed after the experiment. **(F)** Fecal water content was measured daily. Data were expressed as mean ± SD, *p < 0.05, **p < 0.01, ***p < 0.001, ****p < 0.0001, and compared with FC group, *n* = 6.

After establishing a functional constipation model in aging rats, BDF was administered for 14 days. As shown in Figure 1A, at the start, the CON group had significantly higher body weight than the FC group, with the BDF intervention groups showing similar weights to the FC group. Over time, the BDF-H group’s weight increased, surpassing the FC group, while the BDF-L group’s weight remained consistent with the FC group. Figure 1B illustrates daily fecal output, initially similar across all intervention groups and lower than the CON group. With continued BDF intervention, the BDF-H group’s fecal output significantly increased, while the BDF-L group remained comparable to the FC group. Figure 1C shows that the fecal weight-to-body weight ratio in the FC group was half that of the CON group, whereas the BDF-H group nearly doubled this ratio, returning it to the CON group’s level. Figure 1D indicates that the FBST in the FC group nearly doubled compared to the CON group, while the BDF-H group saw a significant 25% decrease. The BDF-L group showed no significant difference from the FC group. A small intestine propulsion test (Figure 1E) revealed that the BDF-H group’s propulsion rate significantly increased, nearing the CON group level. In contrast, the BDF-L group remained similar to the FC group. Figure 1F shows that fecal water content, initially lower in the FC group, increased significantly in the BDF-H group, matching the CON group by day 14. In contrast, the BDF-L group showed no significant change.

**Figure 2:**
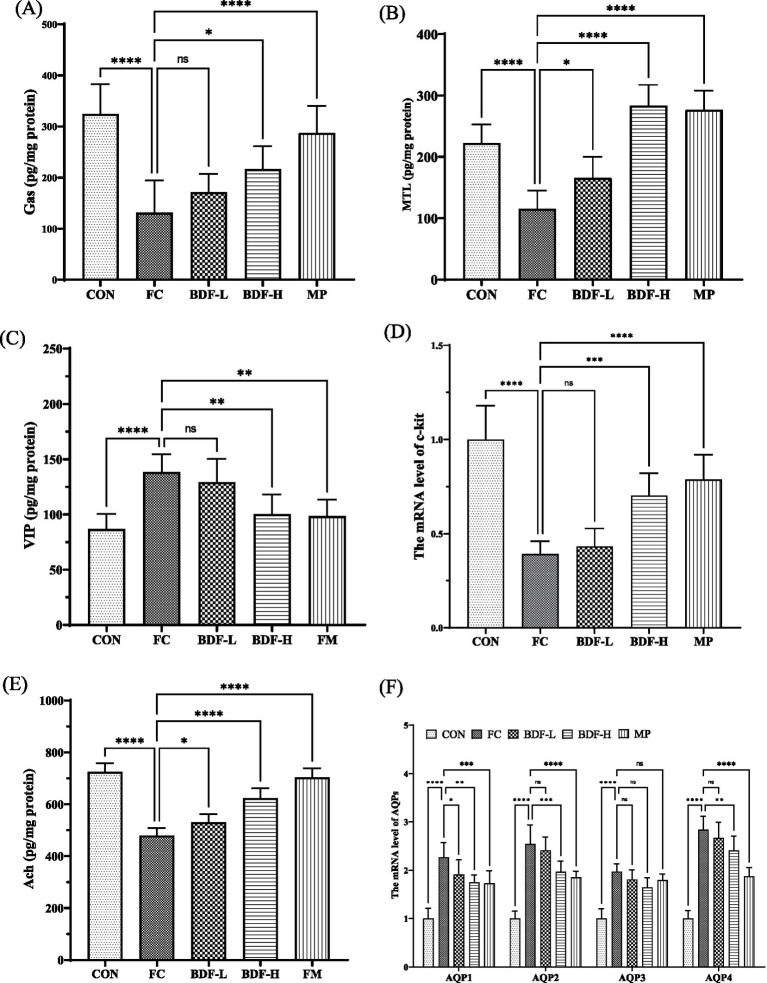
BDF regulated hormones and neurotransmitters associated with constipation. **(A)** Gas, **(B)** MTL, and **(C)** VIP of serum levels that were detected by ELISA. **(D)** mRNA level of c-kit that was detected by quantitative RT-PCR in rat intestinal tissue. **(E)** The content of ACh in rat intestinal tissue was detected by ELISA. **(F)** mRNA levels of AQPs that were detected by quantitative RT-PCR in rat colon tissue. Data were expressed as mean ± SD, *p < 0.05, **p < 0.01, ***p < 0.001, ****p < 0.0001, and compared with FC group, *n* = 6.

After BDF intervention, serum hormones and neurotransmitters were analyzed. Figure 2A shows that the gas levels in the FC group were nearly half of those in the CON group, while the BDF-H group almost doubled the gas levels compared to the FC group. The BDF-L group showed no significant change. As shown in Figure 2B, MTL levels in the FC group were nearly twice those in the CON group, with a substantial increase in the BDF-H group, doubling MTL levels compared to the FC group. The VIP content (Figure 2C) was approximately one-third higher in the FC group than in the CON group but decreased significantly in the BDF-H group. The BDF-L group remained unchanged from the FC group. C-kit mRNA, a marker of ICCs, was analyzed (Figure 2D). In the FC group, c-kit mRNA levels were one-third lower than in the CON group but increased by half in the BDF-H group. The BDF-L group showed no significant changes. Serum acetylcholine (ACh) levels, reduced in the FC group, increased significantly in both BDF groups, with the BDF-H group showing a 20% rise (Figure 2E). Figure 1 shows that fecal water content in aging rats with FC was significantly increased, prompting the examination of aquaporin (AQP) levels related to water reabsorption in intestinal contents. Figure 2F indicates that the mRNA levels of AQPs in the FC group were significantly higher than in the CON group. However, AQP1, AQP2, and AQP4 levels in the BDF-H group showed a significant downward trend compared to the FC group, while AQP2, AQP3, and AQP4 levels in the BDF-L group remained unchanged compared to the FC group.

**Figure 3:**
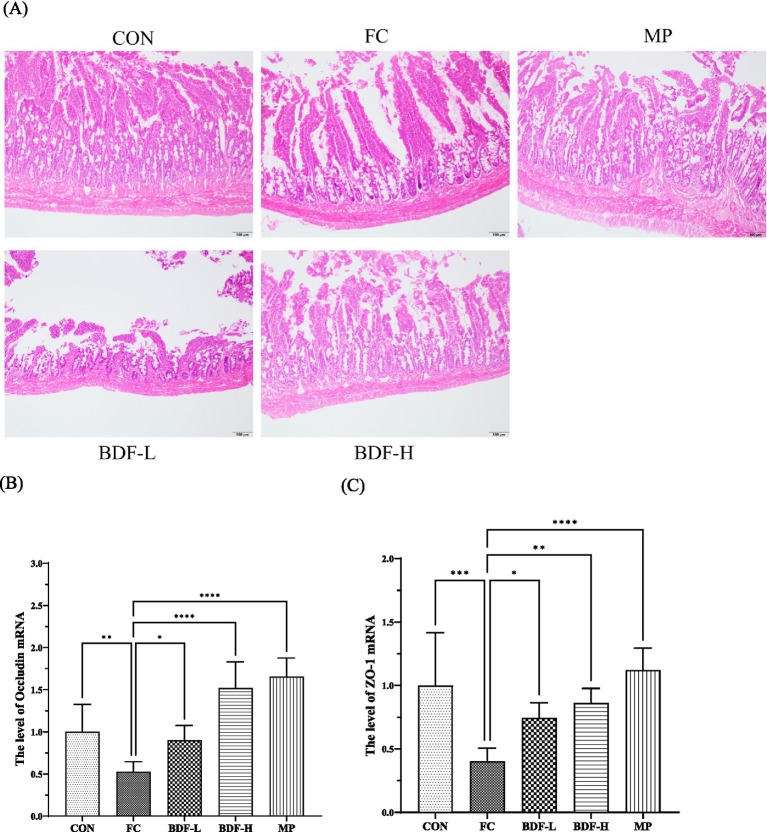
BDF influenced intestinal barrier function in the older rats with FC. **(A)** The small intestine that was stained with H&E, the images presented are with 20× magnification, scale bar: 100 μm. **(B,C)** mRNA levels of occludin, and ZO-1 in the small intestine that were detected by quantitative RT-PCR. Data were expressed as mean ± SD, *p < 0.05, **p < 0.01, ****p < 0.0001, and compared with FC group, *n* = 6.

The hematoxylin and eosin (H&E) staining (Figure 3A) revealed that the CON group had long, intact intestinal villi, closely arranged epithelial cells, and abundant goblet cells. In contrast, aging rats with FC showed damaged villi, increased intercellular space, reduced goblet cells, and multiple inflammatory nodules. The BDF-L group resembled the FC group with sparse, short villi and mucosal damage. However, the BDF-H group exhibited long, intact villi, reduced spacing, increased goblet cells, and minimal inflammatory infiltration. Additionally, occludin and ZO-1 mRNA levels (Figures 3B,C) in the FC group were nearly half of those in the CON group but significantly increased after BDF intervention. In particular, the mRNA level of occludin increased almost two times, and the mRNA level of ZO-1 almost doubled in the BDF-H group, compared with that of the FC group (Figures 3B,C).

**Figure 4:**
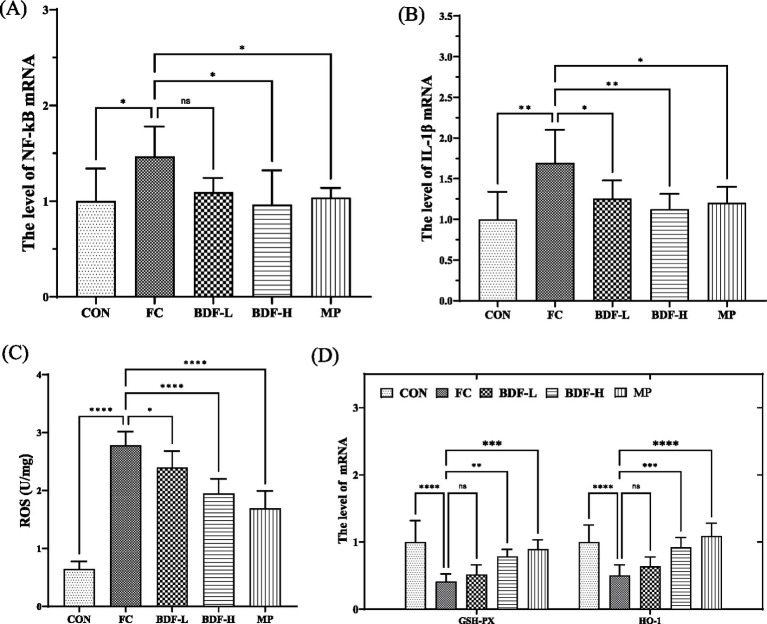
BDF had the potential to inhibit inflammation and intestinal oxidation. **(A,B)** The mRNA levels of NF-κB, IL-1β that were detected by quantitative RT-PCR. **(C)** The levels of ROS in the small intestine that were detected by ELISA. **(D)** The mRNA levels of GSH-Px and HO-1 that were detected by quantitative RT-PCR. Data were expressed as mean ± SD. *p < 0.05, **p < 0.01, ****p < 0.0001, ****p < 0.05 vs. FC group, *n* = 6.

Inflammation and oxidation levels were analyzed across groups. Figures 4A,B shows that nuclear factor-κB (NF-κB) mRNA levels in the FC group were 50% higher than in the CON group but significantly reduced in the BDF-H group. Similarly, interleukin-1-beta (IL-1β) mRNA levels were elevated in the FC group but decreased by approximately one-third in the BDF-H group. Figure 4C indicates that ROS content in the FC group was approximately four times higher than in the CON group, but it was approximately 30% reduction with BDF-H intervention. Figure 4D shows that GSH-Px and HO-1 mRNA levels were 70% lower in the FC group but both nearly doubled after BDF-H treatment.

## 4 Discussion

Currently, aging often relies on medication as the primary intervention for functional constipation. While these drugs are effective, long-term use in aging individuals, whose metabolic function is usually compromised, can lead to drug dependency and resistance (19). In contrast, dietary interventions are healthy and sustainable, with dietary fiber being a recommended nutritional strategy for constipation management.

Functional constipation involves multiple physiological factors, including impaired gastrointestinal motility, hormonal imbalances, and neural regulation. Intestinal motility dysfunction is a key contributor to constipation, leading to prolonged retention of intestinal contents and increased water reabsorption, which results in harder, drier stools (20, 21). Our findings indicate that high doses of BDF significantly improve intestinal motility, increasing the intestinal propulsion rate by approximately one-third and shortening the time to the first black stool by one-quarter, as shown in Figure 1E. Additionally, BDF enhanced fecal water content and increased fecal weight and frequency, suggesting a strong positive correlation between BDF-induced motility and stool hydration. The intestinal motility promoted by BDF was positively correlated with fecal water content; whether there is a regular growth pattern remains to be further verified.

Gastrointestinal hormones and neurotransmitters influence intestinal motility. BDF was found to regulate several key hormones, including an increase in gastrin and motilin while decreasing vasoactive intestinal peptides, thereby stimulating peristalsis and accelerating the transport of intestinal contents, as shown in Figure 2. BDF also elevated acetylcholine levels, further enhancing digestive tract movement and glandular secretion. Interestingly, BDF increased ChAT levels, potentially boosting neurotransmitter synthesis, though it warrants further study. Another critical aspect of intestinal motility is the slow-wave activity generated by interstitial ICCs, where c-kit receptors play a pivotal role (22). The decreased expression of c-kit inhibits the function of ICCs as “pacemakers” for intestinal peristalsis (23). In aging rats with FC, c-kit mRNA expression was significantly reduced, weakening motility. However, BDF treatment notably upregulated c-kit expression, likely due to its protective effects on intestinal wall cells, as BDF also repaired the damaged intestinal barrier in rats. Current research has demonstrated that dietary fiber from various sources can upregulate gastrointestinal hormones and neurotransmitters (24–26). However, limited evidence suggests that dietary fiber can modulate intestinal slow-wave activity. As illustrated in Figure 2D, BDF significantly increases the mRNA levels of c-kit, indicating its potential role in effectively regulating the population of ICCs. It suggests that dietary fiber from burdock root may possess unique advantages distinct from dietary fibers of other origins. Nevertheless, further validation is required to confirm its regulatory effects on slow-wave activity.

For aging people, impaired intestinal function, including mucosal barrier damage and motility disorders, is a significant cause of constipation (27). These issues are interrelated, as slowed motility prolongs exposure to harmful substances, damaging the mucosa, while mucosal damage further impairs motility (28). At the same time, intestinal motility will be affected when the intestinal mucosa is damaged by unbalanced intestinal homeostasis (29). In this study, aging constipated rats showed significant mucosal damage, but dietary fiber intervention repaired the intestinal mucosal layer. The mechanism involves BDF’s anti-inflammatory effects, which reduce NF-κB activation, a key driver of inflammation and mucosal damage (30). BDF also decreased ROS levels and enhanced antioxidant factor expression, likely contributing to reduced inflammation and mucosal barrier repair. We believe that the decreased expression of NF-κB may be related to the reduction of free radicals in intestinal tissues. Studies have found that dietary fiber absorbs intestinal free radicals, which reduces the damage of exogenous free radicals to intestinal tissue (31). Our study found that BDF significantly reduced ROS content in the tissues of aging constipated rats. At the same time, we detected the expression of antioxidant factors such as SOD, GSH, and GSH-Px in rat tissues. We found that BDF could enhance the mRNA expression level of various antioxidant factors. Antioxidant factors are the key enzymes in the body that resist oxidation and remove free radicals. BDF promotes the expression of antioxidant factors, inhibiting inflammation and effectively repairing the intestinal mucosal barrier. The reason BDF promotes the expression of antioxidant factors still needs further exploration.

Although dietary fiber is a well-known natural remedy for constipation, its effectiveness in real-life situations can differ. Some studies report minimal relief in severe cases, possibly due to inadequate dosage (32). One study reported that dietary fiber did not provide significant relief for patients with severe constipation and only provided relief for patients with mild constipation (33). The study did not explore the relationship between the dose and effectiveness of dietary fiber. Our findings suggest that BDF’s anticonstipation effects are dose-dependent, with high doses significantly improving outcomes, including fecal count, water content, and propulsion rate. These results indicated that a daily intake of 35 g/kg body weight of BDF could be an effective intervention for human constipation, although further clinical trials are needed. However, excessive dietary fiber can also have adverse effects, particularly in aging, by forming a gel in the stomach, slowing the food passage, and increasing the risk of bloating and intestinal stress (8). Under normal circumstances, the regulatory effect of dietary fiber on constipation mainly relies on its physical and chemical properties, especially the hydration properties of dietary fiber (8, 34). Therefore, it is crucial to optimize the dosage and administration of BDF to enhance its anticonstipation effects while minimizing side effects. Future research will focus on improving the hydration properties of BDF and its interactions within the gastrointestinal tract to maximize its therapeutic benefits.

In conclusion, high-dose BDF effectively alleviates constipation in aging rats by improving intestinal motility and repairing the mucosal barrier. BDF represents a viable natural functional component for constipation, and appropriate dietary fiber intake is recommended for aging individuals with FC. This study provides insights into dietary interventions for constipation and a theoretical basis for functional foods application in aging people with constipation. In addition, BDF can restore intestinal function through multiple pathways by regulating gastrointestinal hormones, providing anti-inflammatory effects and providing antioxidant effects. This offers new perspectives for personalized treatment of functional constipation in clinical practice.

## Data Availability

The original contributions presented in the study are included in the article/supplementary material, further inquiries can be directed to the corresponding authors.
